# Supplementary material for: The negative association between weight-adjusted-waist index and lung functions: NHANES 2007–2012
Source: PLoS One. 2024 Oct 23;19(10):e0311619. doi: 10.1371/journal.pone.0311619 (PMC11498673; doi:10.1371/journal.pone.0311619)
Supplement: S1 Table — (DOCX) [file pone.0311619.s002.docx]

Table 1 Univariate analysis of variables associated with FVC

| **Variable** | **P value** | **Significant** |
| --- | --- | --- |
| **Age(years)** | <0.05 | Yes |
| **PIR** | <0.05 | Yes |
| **Weight(kg)** | <0.05 | Yes |
| **BMI(kg/m^2^)** | <0.05 | Yes |
| **Waist circumference(cm)** | <0.05 | Yes |
| **HDL (mmol/L)** | <0.05 | Yes |
| **Serum cotinine level (ng/mL)** | <0.05 | Yes |
| **Triglyceride(mmol/L)** | 0.11 | No |
| **LDL(mmol/L)** | <0.05 | Yes |
| **Physical activity (MET-minutes per week)** | <0.05 | Yes |
| **Total Cholesterol (mmol/L)** | <0.05 | Yes |
| **Sex(%)** | <0.05 | Yes |
| **Race** | <0.05 | Yes |
| **Hypertension (%)** | <0.05 | Yes |
| **Had at least 12 alcoholic drinks/1 year? (%)** | <0.05 | Yes |
| **Diabetes mellitus (%)** | <0.05 | Yes |
| **Smoking status (%)** | <0.05 | Yes |
| **Asthma (%)** | <0.05 | Yes |
| **Coronary heart disease (%)** | <0.05 | Yes |
| **Congestive heart failure (%)** | <0.05 | Yes |
| **stroke (%)** | <0.05 | Yes |

Table 2 Univariate analysis of variables associated with FEV1

| **Variable** | **P value** | **Significant** |
| --- | --- | --- |
| **Age(years)** | <0.05 | Yes |
| **PIR** | <0.05 | Yes |
| **Weight(kg)** | <0.05 | Yes |
| **BMI(kg/m^2^)** | <0.05 | Yes |
| **Waist circumference(cm)** | <0.05 | Yes |
| **HDL (mmol/L)** | <0.05 | Yes |
| **Serum cotinine level (ng/mL)** | 0.08 | No |
| **Triglyceride(mmol/L)** | 0.76 | No |
| **LDL(mmol/L)** | <0.05 | Yes |
| **Physical activity (MET-minutes per week)** | <0.05 | Yes |
| **Total Cholesterol (mmol/L)** | <0.05 | Yes |
| **Sex(%)** | <0.05 | Yes |
| **Race** | <0.05 | Yes |
| **Hypertension (%)** | <0.05 | Yes |
| **Had at least 12 alcoholic drinks/1 year? (%)** | <0.05 | Yes |
| **Diabetes mellitus (%)** | <0.05 | Yes |
| **Smoking status (%)** | <0.05 | Yes |
| **Asthma (%)** | <0.05 | Yes |
| **Coronary heart disease (%)** | <0.05 | Yes |
| **Congestive heart failure (%)** | <0.05 | Yes |
| **stroke (%)** | <0.05 | Yes |

Table 3 Univariate analysis of variables associated with FEV1/FVC

| **Variable** | **P value** | **Significant** |
| --- | --- | --- |
| **Age(years)** | <0.05 | Yes |
| **PIR** | <0.05 | Yes |
| **Weight(kg)** | <0.05 | Yes |
| **BMI(kg/m^2^)** | 0.70 | No |
| **Waist circumference(cm)** | <0.05 | Yes |
| **HDL (mmol/L)** | 0.72 | No |
| **Serum cotinine level (ng/mL)** | <0.05 | Yes |
| **Triglyceride(mmol/L)** | <0.05 | Yes |
| **LDL(mmol/L)** | <0.05 | Yes |
| **Physical activity (MET-minutes per week)** | <0.05 | Yes |
| **Total Cholesterol (mmol/L)** | <0.05 | Yes |
| **Sex(%)** | <0.05 | Yes |
| **Race** | <0.05 | Yes |
| **Hypertension (%)** | <0.05 | Yes |
| **Had at least 12 alcoholic drinks/1 year? (%)** | <0.05 | Yes |
| **Diabetes mellitus (%)** | <0.05 | Yes |
| **Smoking status (%)** | <0.05 | Yes |
| **Asthma (%)** | <0.05 | Yes |
| **Coronary heart disease (%)** | <0.05 | Yes |
| **Congestive heart failure (%)** | <0.05 | Yes |
| **stroke (%)** | <0.05 | Yes |

Table 4 Univariate analysis of variables associated with PEF

| **Variable** | **P value** | **Significant** |
| --- | --- | --- |
| **Age(years)** | <0.05 | Yes |
| **PIR** | <0.05 | Yes |
| **Weight(kg)** | <0.05 | Yes |
| **BMI(kg/m^2^)** | <0.05 | Yes |
| **Waist circumference(cm)** | 0.58 | No |
| **HDL (mmol/L)** | <0.05 | Yes |
| **Serum cotinine level (ng/mL)** | 0.10 | No |
| **Triglyceride(mmol/L)** | 0.29 | No |
| **LDL(mmol/L)** | 0.99 | No |
| **Physical activity (MET-minutes per week)** | <0.05 | Yes |
| **Total Cholesterol (mmol/L)** | <0.05 | Yes |
| **Sex(%)** | <0.05 | Yes |
| **Race** | <0.05 | Yes |
| **Hypertension (%)** | <0.05 | Yes |
| **Had at least 12 alcoholic drinks/1 year? (%)** | <0.05 | Yes |
| **Diabetes mellitus (%)** | <0.05 | Yes |
| **Smoking status (%)** | <0.05 | Yes |
| **Asthma (%)** | <0.05 | Yes |
| **Coronary heart disease (%)** | <0.05 | Yes |
| **Congestive heart failure (%)** | <0.05 | Yes |
| **stroke (%)** | <0.05 | Yes |

Table 5 Univariate analysis of variables associated with FEF25%-75%

| **Variable** | **P value** | **Significant** |
| --- | --- | --- |
| **Age(years)** | <0.05 | Yes |
| **PIR** | 0.21 | No |
| **Weight(kg)** | <0.05 | Yes |
| **BMI(kg/m^2^)** | <0.05 | Yes |
| **Waist circumference(cm)** | <0.05 | Yes |
| **HDL (mmol/L)** | <0.05 | Yes |
| **Serum cotinine level (ng/mL)** | <0.05 | Yes |
| **Triglyceride(mmol/L)** | <0.05 | Yes |
| **LDL(mmol/L)** | <0.05 | Yes |
| **Physical activity (MET-minutes per week)** | <0.05 | Yes |
| **Total Cholesterol (mmol/L)** | <0.05 | Yes |
| **Sex(%)** | <0.05 | Yes |
| **Race** | <0.05 | Yes |
| **Hypertension (%)** | <0.05 | Yes |
| **Had at least 12 alcoholic drinks/1 year? (%)** | <0.05 | Yes |
| **Diabetes mellitus (%)** | <0.05 | Yes |
| **Smoking status (%)** | <0.05 | Yes |
| **Asthma (%)** | <0.05 | Yes |
| **Coronary heart disease (%)** | <0.05 | Yes |
| **Congestive heart failure (%)** | <0.05 | Yes |
| **stroke (%)** | <0.05 | Yes |
