# Supplementary material for: The negative association between weight-adjusted-waist index and lung functions: NHANES 2007–2012
Source: PLoS One. 2024 Oct 23;19(10):e0311619. doi: 10.1371/journal.pone.0311619 (PMC11498673; doi:10.1371/journal.pone.0311619)
Supplement: S2 Table — (DOCX) [file pone.0311619.s003.docx]

Table 6 Generalized variance inflation factor (GVIF) analysis results

| **Variable** | **GVIF** | **Df** | **GVIF^(1/(2*Df))** |
| --- | --- | --- | --- |
| **Age(years)** | 1.913627 | 1 | 1.383339 |
| **Race** | 1.438936 | 4 | 1.046538 |
| **PIR** | 1.168726 | 1 | 1.081077 |
| **Weight(kg)** | 8.032244 | 1 | 2.834121 |
| **BMI(kg/m^2^)** | 7.459866 | 1 | 2.731275 |
| **Waist circumference(cm)** | 11.030889 | 1 | 3.321278 |
| **Total Cholesterol (mmol/L)** | 1.918304 | 1 | 1.385028 |
| **Hypertension (%)** | 1.879655 | 1 | 1.371005 |
| **HDL (mmol/L)** | 1.382432 | 1 | 1.175769 |
| **Serum cotinine level (ng/mL)** | 1.757545 | 1 | 1.325724 |
| **Triglyceride(mmol/L)** | 1.187511 | 1 | 1.08973 |
| **LDL(mmol/L)** | 1.654534 | 1 | 1.286287 |
| **Had at least 12 alcoholic drinks/1 year? (%)** | 1.142865 | 1 | 1.069048 |
| **Diabetes mellitus (%)** | 1.21307 | 3 | 1.032716 |
| **Smoking status (%)** | 2.003511 | 2 | 1.189729 |
| **Asthma (%)** | 1.033892 | 1 | 1.016805 |
| **Congestive heart failure (%)** | 1.303734 | 1 | 1.141812 |
| **Coronary heart disease (%)** | 3.35109 | 1 | 1.830598 |
| **stroke (%)** | 3.406149 | 1 | 1.845575 |
| **Physical activity (MET-minutes per week)** | 1.358962 | 1 | 1.165745 |
